# The response of the stem sap flow dynamics of *Tamarix ramosissima* to rainfall variability in the desert-oasis transition zone of Northwest China

**DOI:** 10.3389/fpls.2025.1563166

**Published:** 2025-07-23

**Authors:** Feiyao Liu, Lijuan Zhang, Quangang You, Xian Xue, Guohua Liu, Chang Feng

**Affiliations:** ^1^ College of Geography and Tourism, Hengyang Normal University, Hengyang, China; ^2^ College of Foreign Languages, Hengyang Normal University, Hengyang, China; ^3^ Drylands Salinization Research Station, Northwest Institute of Eco-Environment and Resources, Chinese Academy of Sciences, Minqin, China; ^4^ Key Laboratory of Ecological Safety and Sustainable Development in Arid Lands, Northwest Institute of Eco-Environment and Resources, Chinese Academy of Sciences, Lanzhou, China

**Keywords:** sap flow, phreatophyte xerophytic shrub, desert-oasis transition zone, soil water variability, meteorological factors

## Abstract

Plant transpiration is a fundamental process for maintaining the water cycle, regulating temperature and facilitating nutrient uptake, while also playing a critical role in climate regulation and ecosystem services. However, a significant knowledge gap remains in the understanding of how plant transpiration responds to changes in precipitation patterns within dryland ecosystems. In the present study, the stem sap flow of the phreatophyte xerophytic shrub *Tamarix ramosissima*, meteorological factors, soil moisture content, and bare soil evaporation were examined to assess the effects of two different rainfall categories (category I: lower mean rainfall amount and duration; category II: higher mean rainfall amount and duration) on stem sap flow dynamics. Our results reveal that the rainfall reduced the stem sap flow by 46.5% and 29.5% compared to the previous days across rainfall category I and II, respectively. The daily and diurnal variation of stem sap flow during the three days following rainfall showed non-significant variation compared to pre-rainfall, regardless of rainfall category. The soil moisture content at depth of 0–40 cm (SMC_0-40cm_) exhibits a pronounced increase to rainfall events, irrespective of rainfall category, although these events did not significantly increase the soil available moisture content within this depth. Concurrently, the weighing micro-lysimeters utilized in this study revealed that approximately 91.5% of the total precipitation during the experimental period evaporated into the atmosphere. In addition, the daily stem sap flow on the rainfall day and the following three days post rainfall was strongly positively correlated with photosynthetically active radiation, air temperature, and vapor pressure deficit within the two rainfall categories rather than with SMC_0-40cm_. Together, our findings indicate that the effects of rainfall variability on stem sap flow of *T. ramosissima* are primarily driven by meteorological factors, independent of the rainfall category. The results of this study provide a valuable insight for assessing species-specific water-use strategies and implementing effective reforestation practices in the future.

## 1 Introduction

Climate change impacts water vapor transport and precipitation patterns at global and regional scales, such as rainfall intensity, duration, and intermittency, by altering the thermodynamic distribution characteristics and energy balance of the Earth’s surface and atmosphere (Greve et al., 2014; Novick et al., 2016). Long-term historical observations and modeled scenarios indicate that variability in rainfall patterns will increase (IPCC, 2021). Alterations in precipitation patterns will adversely affect regional hydrological processes, energy balance, and the stability of ecosystems (Donat et al., 2017; Yao et al., 2024). Evapotranspiration, as a key component of regional hydrological processes, couples the water and carbon cycles (Good et al., 2015; Kropp et al., 2017). Arid regions constitute approximately 41% of the total Earth’s land surface, with evaporation accounting for 90-95% of total precipitation, of which vegetation transpiration contributes 40-90% (Ouyang et al., 2021; Smith et al., 2024). Accurately quantifying vegetation transpiration is crucial for understanding the carbon and water cycle in water-limited areas. Rainfall is major driver of biological processes in water limited ecosystem (Sponseller, 2010). And episodic precipitation events of variable timing and frequency can trigger rapid pulses of plant activity (Zhao and Liu, 2010; Chen et al., 2014), thereby enhancing the sensitivity of vegetation to rainfall variability. Thus, elucidating the relationship between transpiration and rainfall variability is essential for understanding the response of plants to climate change in the desert regions.

The stem sap flow is an important indicator of water movement in plants (Wang et al., 2015). Compared to other transpiration monitoring methods (e.g., eddy covariance and lysimeters), sap flow gauges could continuously measure the transpiration and help to mitigate the errors from the allocation of transpiration and evaporation (Hirschi et al., 2017; Perez-Priego et al., 2017; Moorhead et al., 2019). Sap flow responses offer a more precise elucidation of water consumption rates and the tradeoffs in the balance between water supply and demand (Liu et al., 2022; Wang et al., 2022; Liu et al., 2024). The relationship between sap flow and meteorological factors (e.g., photosynthetically active radiation (PAR), water vapor pressure deficit (VPD), air temperature (Ta)) and soil moisture content (SMC) has been analyzed across multiple temporal scales, ranging from diurnal and seasonal dynamics to intra-annual and interannual scales (Zheng and Wang, 2015; Hayat et al., 2020; He et al., 2020; Iqbal et al., 2021; Liu et al., 2022; Su et al., 2022). However, elements of the water cycle in desert plants remain uncertain, particularly with regard to the quantitative understanding of the response to precipitation patterns in the context of climate change. And the response of sap flow dynamics to rainfall may vary on the plant’s habitat and species, water consumption relationships, and functional types within a given area (Zhao and Liu, 2010; Chen et al., 2014; Wang et al., 2020). For example, the sap flow of *Robinia pseudoacacia* increased with precipitation, while non-significant relationship was found between *Quercus liaotungensis Koidz* and precipitation (Du et al., 2011). After 3.8 mm of precipitation, a rapid increase in sap flow was observed in *Nitraria* sp*haerocarpa*, while a more pronounced enhancement in sap flow occurred in *Elaeagnus angustifolia* following 5.2 mm of precipitation (Zhao and Liu, 2010).

*Tamarix ramosissima* is a dominant native phreatophyte xerophytic shrub in the terminal area of the Shiyang River in northwest China. The distribution of lateral fine roots (<2 mm) of *T. ramosissima*, which play a crucial role in water and nutrient uptake, has been found to be primarily concentrated within the top 40 cm of soil (Liu et al., 2022), a layer where SMC are strongly impacted by rainfall. Previous studies have primarily focused on quantifying the transpiration of *T. ramosissima* and examining its controlling factors (Qu et al., 2007; Yu et al., 2017; Liu et al., 2022; Sun et al., 2022). However, the effect of rainfall variability on the sap flow dynamics of phreatophyte xerophytic shrubs is largely unknown. Therefore, it is urgent to explore the response of sap flow dynamics to different rainfall patterns in order to assess whether alterations in rainfall patterns exacerbate the threat to the survival of the *T*. *ramosissima.* Given this concern, the stem sap flow, meteorological factors, soil moisture content, and bare soil evaporation were measured in order to gain a comprehensive understanding of the response of the stem sap flow dynamics of *T*. *ramosissima* to precipitation characteristics during the growing season. The objectives of this study were to: (i) understand the response of stem sap flow of *T*. *ramosissima* to two contrasting rainfall events and associated soil moisture content; (ii) determine the driving factors of daily stem sap flow under two rainfall categories, and (iii) identify the hourly stem sap flow dynamics within different rainfall categories. The results of this study were expected to provide a scientific basis for understanding the mechanisms that underlie the response of desert plants to global climate change.

## 2 Materials and methods

### 2.1 Study site

The study area is located in the downstream of the Shiyang River, which is 60 km from Minqin county. The experiment site is surrounded by Tengger Desert and Badan Jaran Desert, and belong to desert-oasis transition zone. The mean annual air temperature of this region in the past 68 years (1953-2020) is 8.6°C (range from -8.1°C to 23.6°C), and the mean annual precipitation is about 110 mm. The rainfall is concentrated between June to September, accounting for approximately 80% of annual total precipitation. The annual potential evapotranspiration is approximately 1383.0 mm, with a dryness index (ET/P) as high as 12.2. This region has a typical continental desert climate. The groundwater table is approximately 10 m below the surface. And the soil texture is loam (**Supplementary Table S1**).

**Figure 1:**
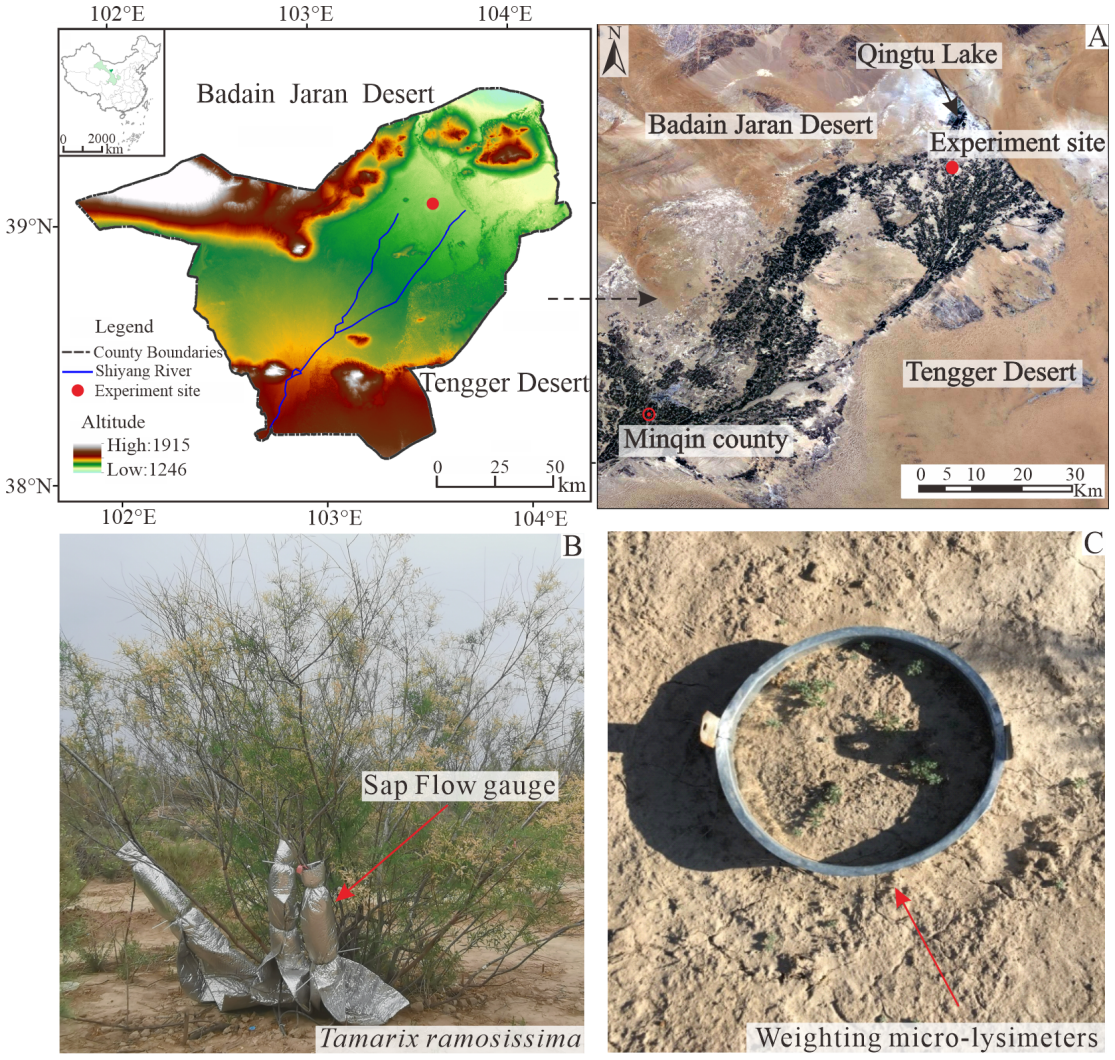
**(A)** Location of the experiment site in the Desert Oasis transition zone of Minqin of China, **(B)** the representative *T*. *ramosissima* shrubs and the equipment for stem sap flow measurements, and **(C)** the picture of weighting micro-lysimeter for bare soil evaporation measurement.

The experiment site was located in the artificial shelter forest-belt in the Desert Oasis transition zone of Minqin of China. The original shelter-belt has been almost completely destroyed as a result of long-term human activities in this area. The existing shelter-belt, established in 2005, exhibits the average coverage of 25% and a stand density of 416 plants per hectare. The dominant shrub species of shelter-belt is *T*. *ramosissima*, with a height ranging from1.5 to 2.5 m. *T*. *ramosissima* has an inverted cone-shaped canopy with no trunk and multiple branches spread obliquely from the base (**Figure 1**). *T*. *ramosissima* exhibits a vertical roots system that can penetrate up to 4 m below ground (Liu et al., 2022). The understory vegetation of *T*. *ramosissima* primarily consist of perennial herbaceous and shrubs, including *Nitraria tangutorum*, *reaumuria songarica, and kalidium foliatum.*

### 2.2 The stem sap flow measurements

We measured the stem sap flow of *T*. *ramosissima* during the growing seasons from May to mid-October of 2020. The stem sap flow gauges (Flow 32, Dynamic Inc., Houston, TX, USA), based on the energy balance method, were used to measure the stem sap flow. Considering the potential positive correlation between stem sap flow and stem cross-sectional area (Wang et al., 2022), and stem diameter was taken as the criteria to measure the stem sap flow. The diameter of 216 stems of *T*. *ramosissima* with 15-years old were investigated before stem sap flow gauges equipment. The results showed that the stem with diameter of 10–20 mm and 20–25 mm accounted for 60.2% and 17.6% of the total investigated diameter of the 15-years old *T*. *ramosissima*, respectively (**Table 1**). In order to accurately capture the response of stem sap flow dynamics to rainfall variability, six stems with different diameters were selected. The selected stems exhibiting optimal growth conditions and free from visible pest or disease infestations were selected for measurement of stem sap flow. The canopy area, height, and basal diameter of six selected stems were obtained using a steel tape measure with 1 mm resolution. And the diameters of these selected stems were 10.2 mm, 14.3 mm, 11.8 mm, 18.8 mm, 22.0 mm and 31.2 mm (**Table 2**). Six gauges with five models were deployed to measure stem sap flow, including two gauges of model for 11 mm (SGEX9), one gauge of each model for 14 mm (SGEX13), 18 mm (SGEX13), 22 mm (SGEX19), and 31 mm (SGEX25). Therefore, the sap flow of selected stems could represent the stem sap flow characteristics of the 15-years old *T*. *ramosissima.*

**Table 1:** The characteristics of investigated 216 stems of *T. ramosissima* with 15-years old of age.

Diameter classification (mm)	Ranges	Average diameter (mm)	Frequency	Relative frequency (%)
0<D<10mm	7-9.75	8.5	16	7.4
10≤D<15mm	10-14.97	12.6	65	30.1
15≤D<20mm	15-19.75	17.3	65	30.1
20≤D<25mm	20-24.8	22.3	38	17.6
25≤D<30mm	25.2-29.6	26.5	16	7.4
30≤D<35mm	30.5-33.9	32.2	10	4.6
35≤D<40mm	35.4-37.6	36.2	5	2.31
40≤D<45mm	–	–	0	0
45≤D ≤ 50mm	50	50	1	0.5

“-” indicate that no stems with a diameter between 40 mm and 45 mm were observed in this investigation.

**Table 2:** The summary morphological characteristics of selected stems of *T*. *ramosissima*.

Stem number	1	2	3	4	5	6
Height (cm)	124	145	152	157	165	178
Stem diameter (mm)	10.2	14.3	11.8	18.8	22.0	31.2

All selected branches were in good condition and able to support the weight of the stem sap flow gauge throughout the experimental periods. Gauges were carefully installed following the manufacturer’s recommendations, and more detailed information on the installation can be found in (Liu et al., 2022). After the installation of the stem sap flow gauges, the heater input voltage was monitored and adjusted according to the sensor voltage requirements. To minimize the risk of overwriting previously recorded data arising from the large volume of incoming signals, the output from the gauges was sampled at 10-second intervals and stored as 30-minute mean values using a CR1000 datalogger (Campbell Scientific, Logan, USA). The methodology for calculating stem sap flow of *T*. *ramosissima* is detailed in (Wang et al., 2020; Liu et al., 2022).

### 2.3 Meteorology and soil moisture content measurements

In the experiment site, a TE525 bucket rainfall gauge (Campbell Scientific, Inc, USA) (0.1 mm per tip) was installed to record the precipitation amount and duration, and rainfall intensity (mm·h^-1^) could be obtained accordingly. During the experimental period, rainfall events were recorded on 24 days, of which 6 rainfall events with precipitation less than 1 mm. The accumulation precipitation in the experimental period was 63.8 mm, the longest rainfall duration and highest rainfall intensify were 10.5 h and 2.2 mm·h^-1^, respectively.

Considering the effects of gully micro-geomorphology, an automatic weather station was established to monitor micrometeorological factors. Air temperature (Ta), relative humidity (RH) (HMP155A, Campbell Scientific, Logan, USA), wind speed and wind direction (WS and SD) (WindSonic75, Gill Instruments Limited, UK) were observed at 2.5 m above the soil surface, respectively. The photosynthetically active radiation (PAR) at the height of 2.5 m from the soil surface were monitored by PQS1 (PQS1, Kipp & Zonen, Inc). All the data signals were recorded at 10 s intervals and stored as 30 min averages using a CR3000 datalogger (Campbell Scientific, Logan, USA). The daily Ta, daily RH, daily WS and mean daily PAR in this study are the average values from 00:00 to 24:00 (local time), respectively. The vapor pressure deficit (VPD) was calculated with RH and Ta using the method described in (Cambell and Norman, 1998).

In the experiment site, the volumetric soil moisture (SMC, cm^3^·cm^-3^) was monitored along with five replicates located outside the canopy of selected *T*. *ramosissima*, where stem sap flow measurements were also conducted. The 5TM soil probes (Campbell Scientific, Inc, USA) were buried at six depths below the soil surface (5 cm, 10 cm, 20 cm, 40 cm, 80 cm, and 160 cm). The data were recorded at 10 s intervals and stored as 30 min average values with a CR3000 datalogger (Campbell Scientific, Logan, USA). The monitored soil moisture content data was calibrated and corrected with the gravimetric method every month. Soil relative extractable water (REW) was calculated as follows (Equation 1) (Granier, 1987):


(1) $$REW=\frac{\theta -\theta_{wp}}{\theta_{fc}-\theta_{wp}}$$

where *θ* is the monitored volumetric soil moisture content, *θ_fc_* (cm^3^·cm^-3^) and *θ_wp_* (cm^3^·cm^-3^) are soil water content at field capacity and wilting point. The *θ_fc_* and *θ_wp_* were derived from the soil-water characteristic curve. The wilting point was considered to be 0.12 cm^3^·cm^-3^, based on the method described in (Wiecheteck et al., 2020).

### 2.4 Bare soil evaporation measurements

Soil evaporation was measured using 14 sets of weighting micro-lysimeters (**Figure 1C**). We selected seven typical *T*. *ramosissima* within the experimental stand and two weighting micro-lysimeters adjacent to each selected shrub, one below the canopy and the other in the gap between shrub. The weighting micro-lysimeters consist of a weighting system and lysimeter vessel comprising a stainless steel cylinder with a diameter of 25 cm and a height of 50 cm. The weight of micro-lysimeters weighted at 08:00 AM and 20:00 PM daily during the experimental period using the SP30001 electronic scales with an accuracy and range of 0.5 g and 100 kg, respectively. The evaporation of bare soil was calculated according the water balance method, as follow (Equation 2) (Lyles et al., 2024):


(2) $$E=\frac{(G_{1}-G_{2})}{A}\times 10+P-D$$

where E is the bare soil evaporation (mm), when it is negative, it indicates the presence of condensate water, G_1_ And G_2_ are the mass measured at t_1_ and t_2_, A is the cross-sectional are of weighting micro-lysimeters (cm^2^), 10 is the conversion, P is the rainfall amount from t_1_ to t_2_ (mm), D is the drainage of same period (mm).

### 2.5 Classification of rainfall category

The 18 rainfall events during the experimental period in 2020 were classified into two rainfall categories based on rainfall amount and rainfall duration using the *K*-means clustering (**Table 3**). Starting from these cluster centres, the rainfall events are selected into two cluster in the way that keep the overall within-group variance at a minimum. Attempts were made until the most suitable clusters appeared and normally, and the classification must meet the t-test criterion of significant level (*P*<0.05) (Wang et al., 2020).

**Table 3:** Statistics information of different rainfall categories during the experimental period in 2020.

Rainfall category	Rainfall characteristics	Ranges	Mean ± standard deviation	Accumulated rainfall (mm)	Times
I	Rainfall amount (mm)	0.1-3.9	1.59 ± 0.22a	27.8	13
	Rainfall duration (h)	0.5-5	2.02 ± 0.29a		
	Rainfall intensity (mm·h^-1^)	0.2-3.9	0.75 ± 0.48a		
II	Rainfall amount (mm)	5.3-11.3	7.2 ± 0.46b	36.0	5
	Rainfall duration (h)	3-10.5	4.75 ± 1.48b		
	Rainfall intensity (mm·h^-1^)	0.9-1.8	1.33 ± 0.58a		

The different letters indicate significant difference of rainfall amount and rainfall duration (t-test, *P*<0.05). The average values mentioned above are expressed as the means ± standard error.

As presented in **Table 3**, rainfall category I has the lower mean value of rainfall amount and rainfall duration (1.59 ± 0.22 mm and 2.02 ± 0.29 h, respectively). In contrast, rainfall category II is associated with higher mean values for rainfall amount and duration (7.2 ± 0.46 mm and 4.75 ± 1.48 h, respectively). And there is non-significant difference in rainfall intensity between rainfall category I and rainfall category II. During the experimental period, rainfall events occurred with a frequency of 72.2% for category I and 27.8% for rainfall category II. The accumulated rainfall amount of rainfall category I and rainfall category II were 27.8 mm and 36.0 mm, respectively.

### 2.6 Date processing and analysis

To eliminate the effect of the large variations in stem sap flow, the half-hourly stem sap flow data presented in this study is the average values for all six selected stems. Daily stem sap flow was the sum of half-hourly stem sap flow in a day. Given that this study focuses on only two rainfall categories, t-test was employed to assess the differences in meteorological factors, SMC and REW at different soil layers during the two rainfall categories. ANOVA was performed to evaluate the statistical differences in daily stem sap flow and meteorological factors across pre-rainfall, rainfall, and post-rainfall periods within the same category. The correlation test and linear regression fitting were employed to explore the relationship between environmental factors and stem sap flow in rainfall day and post-rainfall for two rainfall categories. The level of significance was set at 95% confidence interval (*P*=0.05). All statistics analyses were performed with SPSS 22 (SPSS Inc., USA).

Structural equation modeling (SEM) was employed to quantify both the direct and indirect relationships between environmental factors affecting the stem sap flow of *T. ramosissima* on the rainfall day and the subsequent three days. A conceptual model was developed based on the correlation analysis between the forcing variables and the response variable. All variables significantly correlated with stem sap flow were incorporated into the base model. Non-significant relationships were removed during model refinement using modification indices. Model coefficients were estimated via the maximum-likelihood method. When lower value of chi-square (χ^2^, ≤2) and root mean square error of approximation (*RMSEA*, ≤0.05), and higher *P*-value (*P*<0.05) suggest that the overall goodness of model fit (Schermelleh-Engel et al., 2003). SEM analysis was performed using AMOS 26.0 (SPSS Inc., USA).

## 3 Results

### 3.1 Variation in environmental factors and bare soil evaporation

**Figure 2:**
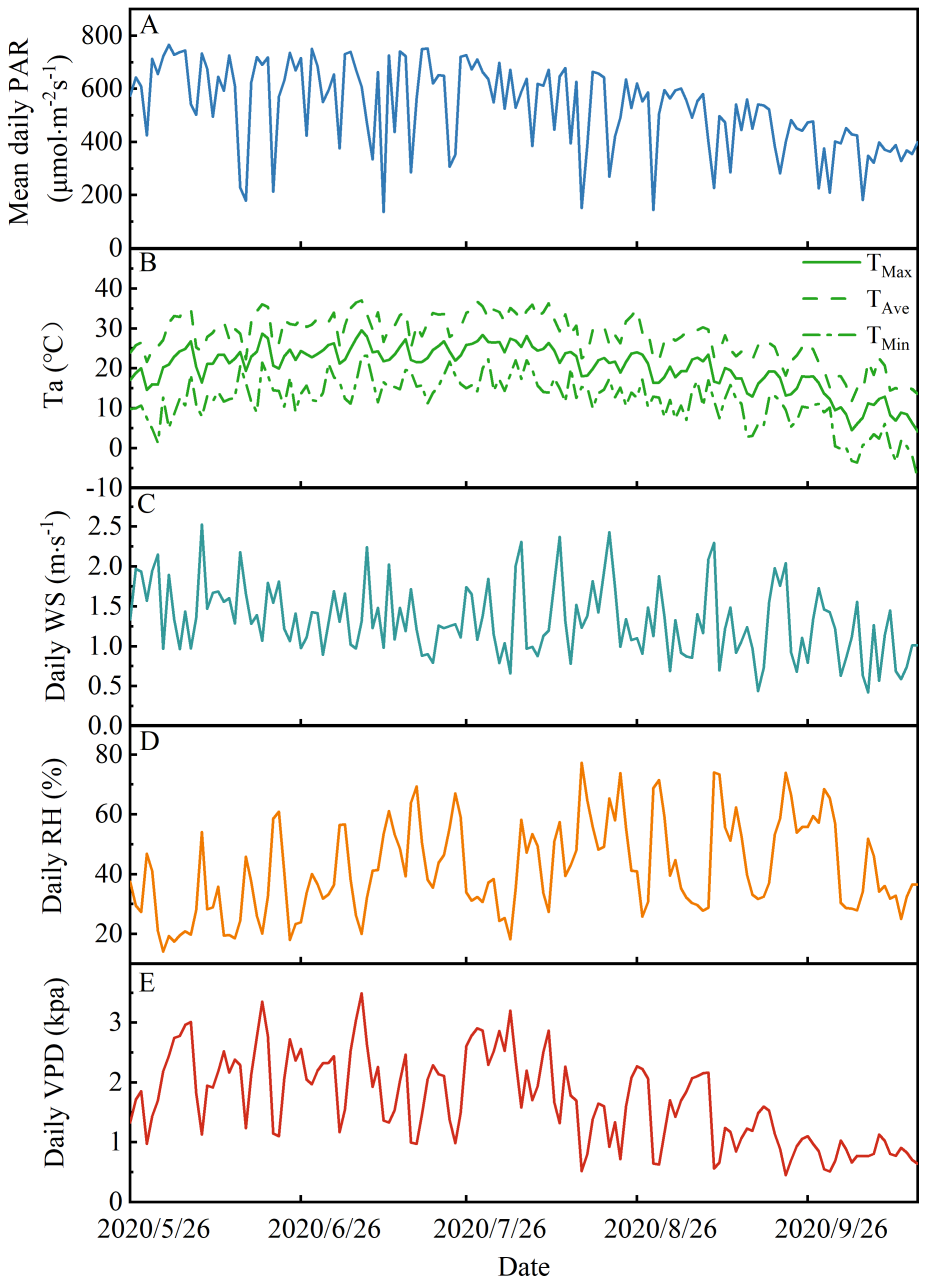
Changes in meteorological factors during the experimental period in 2020. **(A)** Mean daily (average value from 00:00 to 24:00, local time) photosynthetically active radiation (PAR), **(B)** maximum temperature, average air temperature, and minimum temperature (T_Max_, T_Average_ and T_Min_), **(C)** daily wind speed (WS), **(D)** daily relative humidity (RH), and **(E)** daily vapor pressure deficit (VPD) during the experimental period in 2020.

The temporal variations in mean daily PAR, Ta, Ta_Max_, Ta_Min_, RH, and VPD during the study period in 2020 are presented in **Figure 2** and **Supplementary Figure S1**. These variables exhibited distinct seasonal patterns throughout the experimental period. Specifically, mean daily PAR, Ta, RH, and VPD showed pronounced seasonal fluctuations. The ranges of these variables during the study period were as follows: mean daily PAR ranged from 137.2 to 765.7 μmol·m²·s^−1^(**Supplementary Figure S2**), Ta ranged from 20.0 to 29.6°C, Ta_Max_ ranged from 11.84 to 37.07°C, Ta_Min_ ranged from -7.85 to 23.21°C, RH ranged from 13.2% to 76.5%, WS ranged from 0.4 to 2.5 m·s^−1^, and VPD ranged from 0.5 to 3.5 kPa. The highest monthly mean values for PAR and VPD were observed in June; in comparison, the peak monthly mean values for Ta and RH occurred in July (24.79°C) and September (49.36%), respectively.

**Figure 3:**
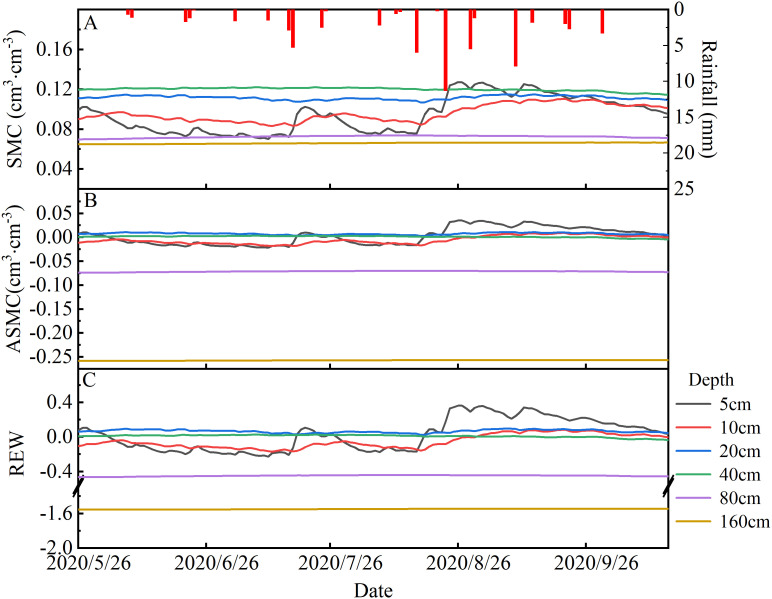
Daily variation of the rainfall and **(A)** soil moisture content (SMC), **(B)** available soil moisture content (ASMC), and **(C)** soil relative extractable water (REW) during the experimental period in 2020.

The SMC in 0–40 cm of the soil profile was more responsive to rainfall, with a delayed response to heavy rainfall and generally remains stable at 40–160 cm (**Figure 3A**). Therefore, the depths of 0–40 cm and 40–160 cm were classified as shallow (SMC_0-40cm_) and middle (SMC_40-160cm_) soil layers, respectively. Daily SMC_0-40cm_ and daily available SMC_0-40cm_ ranged from 0.10 to 0.12 cm^3^·cm^-3^ and from -0.01 to 0.01 cm^3^·cm^-3^ during the experimental period, respectively. In comparison, daily SMC_40-160cm_ and daily available SMC_40-160cm_ remained constant, recorded at 0.07 cm^3^·cm^-3^ and -0. 13 cm^3^·cm^-3^ during the study period (**Figure 3B**). Daily REW at 0–40 cm and 40–160 cm soil layers ranged from -0.08 to 0.13, and -1.01 to -0.99, respectively (**Figure 3C**).

The statistical information of average daily meteorological factors and daily maximum meteorological factors among two rainfall categories is shown in **Table 4** and **Supplementary Table S2**. And mean daily PAR, Ta, RH, and VPD ranged from 137.2 to 765.7 μmol·m^-2^s^-1^, from 4.2 to 28.6 °C, from 14.1 to 77.3%, and from 0.5 to 2.5 kPa, respectively, across the two different rainfall categories. On rainfall days, the mean daily RH and VPD were significantly higher in rainfall category II than in category I (*P*< 0.05), with non-significant differences observed for mean daily PAR and Ta between the two rainfall categories (*P*> 0.05). A significant difference in mean daily RH and PAR was observed between the rainfall day and the subsequent three days (*P*< 0.05). However, daily Ta did not exhibit significant variation within this category (*P*>0.05). Relative to rainfall days, RH showed a significant decline, whereas VPD exhibited a significant increase during the three days following rainfall events (*P*<0.05). Conversely, non-significant differences in mean daily PAR and Ta were detected among the two rainfall categories (*P* > 0.05).

**Table 4:** Statistical information of mean daily meteorological factors among two rainfall categories during the experimental period in 2020.

Rainfall category	Time	PAR (μmol·m^-2^s^-1^)	Ta (°C)	RH (%)	VPD (kPa)
Category I	PR	519.2 ± 51.7abA	23.3 ± 1.2aA	45.4 ± 3.9abA	1.9 ± 0.2bA
	RD	401.8 ± 39.5aA	19.9 ± 1.7aA	59.2 ± 3.8bA	1.1 ± 0.2aA
	FDAR	600.4 ± 67.9bA	20.5 ± 2.3aA	50.0 ± 7.5abA	1.6 ± 0.2bA
	SDAR	556.4 ± 33.2abA	20.5 ± 1.3aA	41.5 ± 3.6aA	1.7 ± 0.2bA
	TDAR	623.9 ± 29.3bA	21.4 ± 1.5aA	38.4 ± 3.0aA	2.0 ± 0.2bA
Category II	PR	464.3 ± 56.2abA	22.3 ± 0.4aA	45.9 ± 6.3abA	1.7 ± 0.2bA
	RD	315.5 ± 79.2aA	18.3 ± 1.4aA	72.7 ± 2.8cB	0.7 ± 0.1aB
	FDAR	556.1 ± 71.1abA	18.9 ± 1.8aA	63.1 ± 6.2bcA	1.0 ± 0.3abA
	SDAR	602.8 ± 43.9bA	20.8 ± 0.9aA	50.1 ± 3.8abA	1.6 ± 0.2bA
	TDAR	549.7 ± 60.6abA	22.1 ± 0.9aA	43.1 ± 2.6aA	1.8 ± 0.2bA

PR, RD, FDAR, SDAR, and TDAR are pre-rainfall, the rainfall day, the first day after rainfall, the second day after rainfall, and the third day after rainfall. The average values mentioned above are expressed as the means ± standard error. Different upper-case letters indicate significant differences in mean daily meteorological factors on the same day among the different rainfall categories (t-test, *P*<0.05). Different lower-case letters indicate significant differences in mean daily meteorological factors among pre-rainfall, rainfall and pos-rainfall day in the same rainfall category (ANOVA, *P*<0.05).

Compared to the pre-rainfall day, there was non-significant increase in SMC, available SMC, or REW values at 0–40 cm and 40–160 cm soil layers on the rainfall day or the subsequent three days across both categories (*P*>0.05). Throughout the rainfall day and the following three days in rainfall category I, the average daily SMC at depths of 0–40 cm and 40–160 cm remained unchanged relative to pre-precipitation levels.

Furthermore, the results revealed that the available SMC at 0–40 cm and 40–160 cm soil layers was 0.00 cm³·cm^−3^ and -0.13 cm³·cm^−3^, respectively, indicating that the soil may unable to provide adequate moisture for plant uptake during this period. In rainfall category II, the daily mean SMC at 0–40 cm and 40–160 cm soil layers increased from 0.11 cm³·cm^−3^ and 0.07 cm³·cm^−3^ on pre-rainfall day to 1.65 cm³·cm^−3^ and 0.11 cm³·cm^−3^ on the first day following rainfall events. Nevertheless, the available SMC in these layers remained at 0.00 cm³·cm^−3^ and -0.13 cm³·cm^−3^, respectively. These findings suggest that the average daily SMC remained in a state of deficiency irrespective of the soil layer or rainfall category.

Concurrently, the weighing micro-lysimeters showed that the evaporation of bare soil was 58.4 ± 1.2 mm, accounting for around 91.5% of the total precipitation during the experimental period.

### 3.2 Dynamics of daily stem sap flow within two rainfall categories

**Figure 4:**
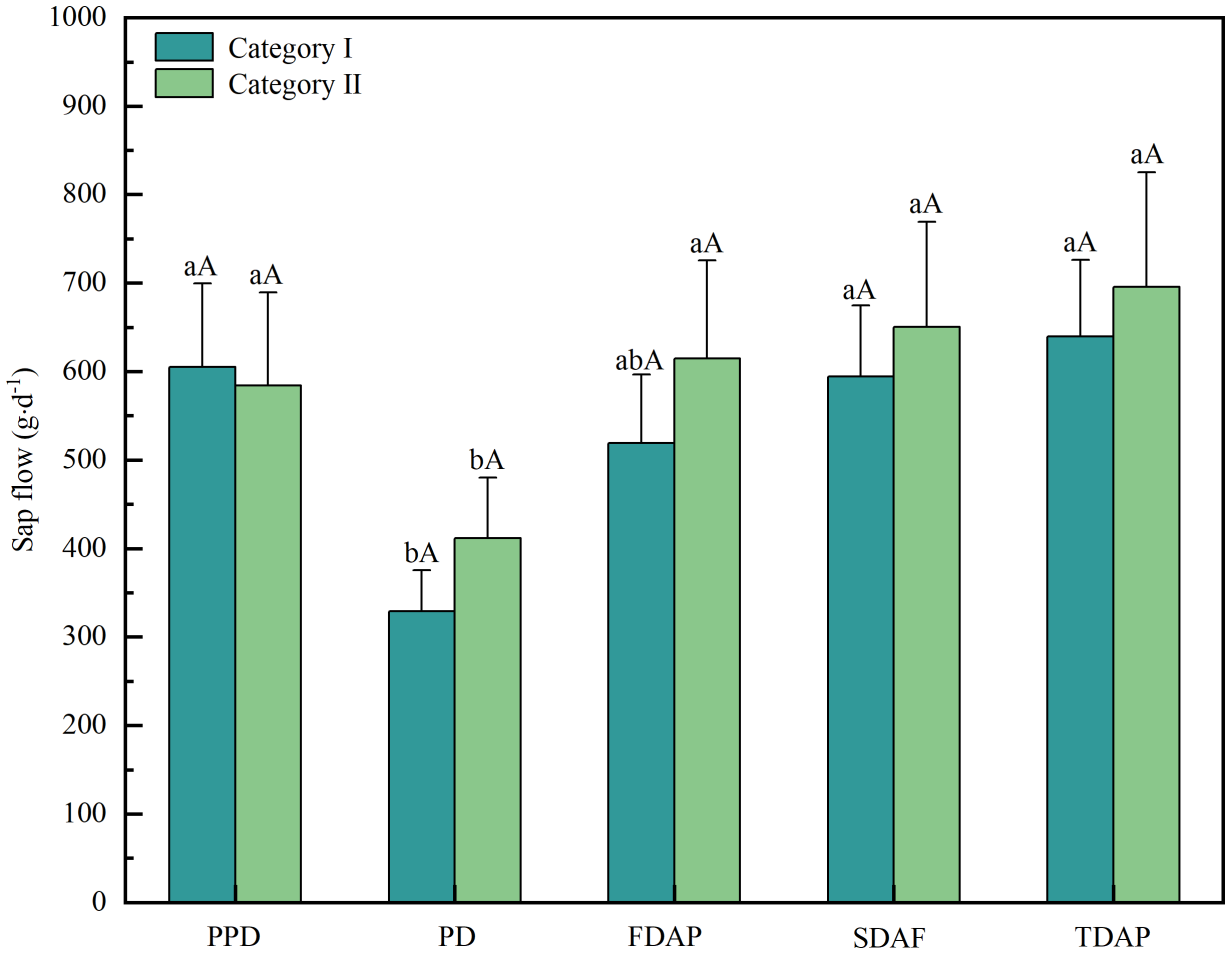
Comparison of the mean daily stem sap flow among the pre-rainfall, rainfall, and post-rainfall periods among different rainfall categories during the experimental period in 2020 for *T. ramosissima*. PR, RD, FDAR, SDAR, and TDAR refer to pre-rainfall, the rainfall day, the first day after rainfall, the second day after rainfall, and the third day after rainfall, respectively. Different uppercase letters denote significant differences in daily stem sap flow on the same day among category I and category II (*P*<0.05). Different lowercase letters signify significant differences in daily stem sap flow among the pre-rainfall, rainfall, and post-rainfall periods within the same rainfall category (*P*<0.05).

Daily stem sap flow of *T*. *ramosissima* was classified based on the rainfall classification during the study period of 2020. There were 13 and 8 daily stem sap flow data samples in rainfall category I and rainfall category II, respectively. The daily stem sap flow of *T*. *ramosissima* ranged from 265.9 to 535.1 g·d^-1^ in rainfall day of category I, and from 167.9 to 636.9 g·d^-1^ in rainfall day of category II. Moreover, the mean daily stem sap flow of *T*. *ramosissima* was 329.2 ± 46.1 g·d^-1^ and 411.8 ± 68.1 g·d^-1^ on the rainfall day in category I and rainfall category II, respectively (**Figure 4**). Non-significant difference in the mean daily stem sap flow was observed between the two rainfall categories (*P*>0.05).

Non-significant difference was observed in mean daily sap flow on pre-rainfall day between the two rainfall categories (*P*>0.05). The average daily stem sap flow of *T*. *ramosissima* on pre-rainfall day was 605.0 ± 94.8 g·d^−1^ for rainfall category I and 584.0 ± 105.0 g·d^−1^ for rainfall category II. The occurrence of rainfall events significantly decreased daily stem sap flow in both rainfall categories (*P*<0.05). Specifically, compared with the daily stem sap flow on pre-rainfall day, the average daily stem sap flow of *T. ramosissima* decreased by 45.6% in rainfall category I and by 29.5% in rainfall category II. Furthermore, non-significant difference was found in mean daily stem sap flow on pre-rainfall day between the two rainfall categories (*P*>0.05).

Compared to the daily stem sap flow of the pre-rainfall day, non-significant increase was observed in the following three days after rainfall in both rainfall category I and rainfall category II (*P*>0.05). On the first, second, and third days post-rainfall, the ranges of average daily stem sap flow for rainfall category I and rainfall category II were 319.5-846.4 g·d^−1^ and 223.4-970.9 g·d^−1^, 327.9-897.9 g·d^−1^ and 232.5-1018.6 g·d^−1^, and 365.0-979.7 g·d^−1^ and 242.2-1098.2 g·d^−1^, respectively. The mean daily stem sap flow of *T*. *ramosissima* on the first, second, and third day after rainfall were 519.2 ± 77.6 g·d^-1^, 594.3 ± 80.0 g·d^-1^, and 639.41 ± 86.5 g·d^-1^ in rainfall category I, respectively. In rainfall category II, the corresponding values were 615.0 ± 110.7 g·d^−1^, 650.4 ± 118.5 g·d^−1^, and 695.3 ± 129.7 g·d^−1^. Compared to the daily stem sap flow of rainfall day, the daily stem sap flow of *T. ramosissima* in the subsequent three days of rainfall have significant increase in both category I and category II (*P*< 0.05). However, non-significant differences were detected in daily stem sap flow of *T*. *ramosissima* between the two rainfall categories on the same day after rainfall (*P*>0.05). Meanwhile, the daily maximum sap flow has been compared among the pre-rainfall, rainfall, and post-rainfall periods among different rainfall categories (**Supplementary Figure S3**). Compared to the daily maximum sap flow of the pre-rainfall day, non-significant increase was observed in the following three days after rainfall in both rainfall category I and rainfall category II (*P*>0.05). Moreover, non-significant differences in maximum sap flow of *T*. *ramosissima* between the two rainfall categories on the same day post-rainfall was observed(*P*>0.05).

### 3.3 Response of daily stem sap flow to environmental factors within different rainfall categories

The daily stem sap flow of rainfall day and the following three days after rainfall are affected by different factors in two rainfall categories (**Table 5**). The daily stem sap flow on rainfall day and the subsequent three days throughout the study period exhibited strong correlations with mean daily PAR, Ta, RH, and VPD in both rainfall categories (*P*<0.01). In contrast, non-significant correlation was observed between daily stem sap flow and daily SMC in the 40–160 cm soil layer, regardless of the rainfall category (*P*>0.05). Furthermore, the correlation analysis revealed a significant negative relationship between daily stem sap flow and SMC in the 0–40 cm soil layer on the rainfall day and subsequent three days in rainfall category I. However, no such correlation was observed in rainfall category II.

**Table 5:** Correlation coefficients between daily rainfall stem sap flow of *T*. *ramosissima* and environmental factors under two rainfall categories among the experimental period in 2020.

Rainfall category	PAR	Ta	Ta_Max_	Ta_Min_	RH	VPD	SMC_0-40cm_	SMC_40-160cm_	REW_0-40cm_	REW_40-160cm_
Category I	0.77^**^	0.71^**^	0.82^**^	0.30^**^	-0.45^**^	0.78^**^	-0.39^**^	0.07	-0.39	0.56^**^
Category II	0.90^**^	0.79^**^	0.84^**^	-0.18	-0.87^**^	0.88^**^	-0.04	-0.39	-0.03	-0.02

PAR, Ta, Ta_Max_, Ta_Min_, RH, and VPD are photosynthetically active radiation, average daily air temperature, maximum daily air temperature, minimum daily air temperature, relative humidity and vapor pressure deficit. SMC and REW are short for soil moisture content and relative extractable water. ^**^ *P*<0.01.

**Figure 5:**
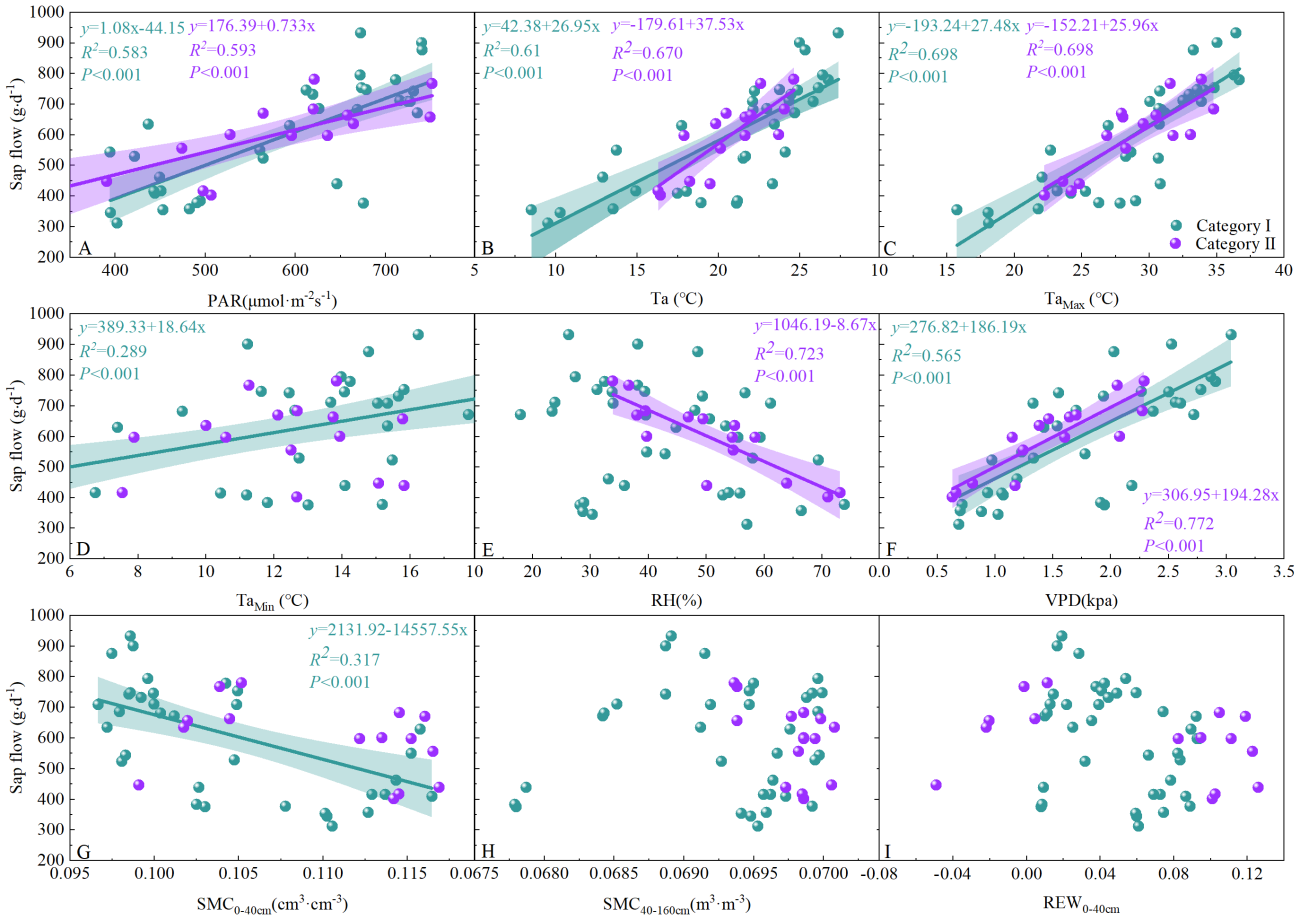
Relationship between daily stem sap flow of *T. ramosissima* and mean daily PAR, daily Ta, daily Ta_max_, daily Ta_min_, daily RH, daily VPD, SMC_0-40cm_, SMC_40-160cm_ and REW_0-40cm_ within the different rainfall categories during the experimental period in 2020.

To assess the potential lag effects of rainfall on the stem sap flow of *T. ramosissima*, the relationship between daily stem sap flow and environmental factors was analyzed separately for the three days following rainfall. The linear relationships between daily stem sap flow and mean daily PAR, mean daily Ta, daily maximum air temperature (Ta_Max_), daily minimum air temperature (Ta_Min_), daily RH, daily VPD, daily SMC, and daily REW_0-40cm_ of the three days following rainfall within the two rainfall categories are shown in **Figure 5**. Daily stem sap flow of the three days following rainfall was positively correlated with increases in mean daily PAR, mean Ta, Ta_Max_, Ta_Min_, and VPD among the two rainfall categories. In contrast, daily stem sap flow decreased with increasing RH in both rainfall categories. Unexpectedly, with the increase in SMC_0–40 cm_, the daily stem sap flow of the three days following rainfall showed a significantly decline in rainfall category I. Furthermore, the relationship between daily maximum stem sap flow and daily maximum PAR, mean daily Ta, daily maximum Ta, daily minimum Ta, daily minimum daily RH, daily maximum VPD, SMC_5cm_, and SMC_10cm_ for *T. ramosissima* in the following three days of rainfall events of two rainfall categories during the experimental period in 2020 have been explored (**Supplementary Figure S4**). Daily maximum sap flow over the three days following rainfall was positively correlated with daily maximum PAR, daily mean Ta, daily minimum Ta, and daily maximum VPD across two rainfall categories. The daily maximum sap flow over the three days following rainfall was negatively with daily minimum RH, SMC_5cm_ and SMC_10cm_.

**Figure 6:**
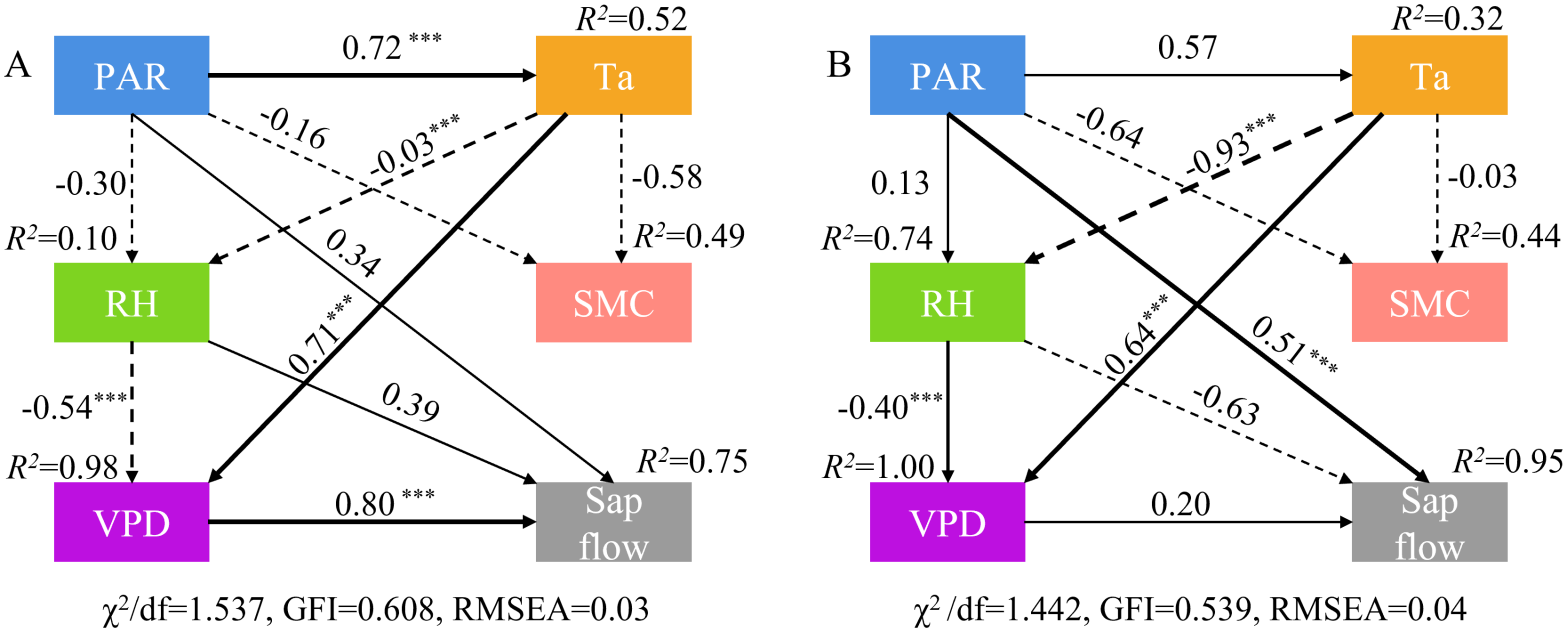
Structural equation models showing the relationships among PAR, Ta, RH, SMC and daily stem sap flow for *T. ramosissima* in the rainfall day and the following three days of the category I **(A)** and category II **(B)**. The value beside each arrow represents the standardized path coefficient. The thickness of the arrow is proportional to the standardized path coefficient on the lines, where solid lines indicate positive values and dashed lines indicate negative values. ***P<0.001.

In our structural equation models, mean daily PAR, daily Ta, daily RH, and daily VPD explained 75% and 95% of the variance in daily stem sap flow for rainfall categories I and II, respectively (**Figure 6**). VPD had a significant direct impact on daily stem sap flow within category I (*P*<0.001); however, there was non-significant direct impact on daily stem sap flow within category II (*P*>0.05). PAR indirectly affects stem sap flow through Ta and VPD in rainfall category I. Additionally, PAR showed a significant direct positive association with stem sap flow (*P*< 0.001). And VPD exhibited the highest standardized total effect on stem sap flow in rainfall category I; in contrast, PAR demonstrated the highest standardized total effect on stem sap flow in rainfall category II. Notably, SMC had no direct or indirect impact on stem sap flow within the two rainfall categories during the study period.

### 3.4 Half-hour stem sap flow dynamics within two different rainfall categories

**Figure 7:**
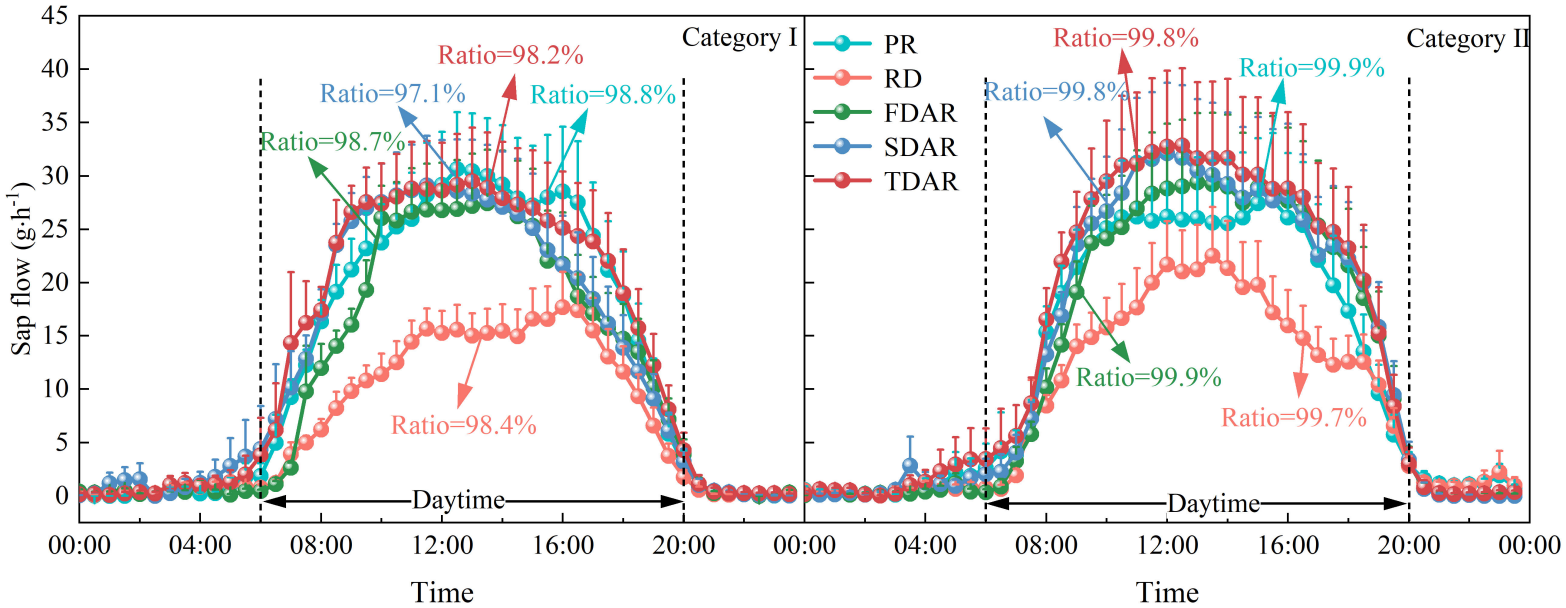
Diurnal variation in mean half-hourly stem sap flow in pre-rainfall, rainfall, and post-rainfall days within the two rainfall categories during the experimental period in 2020 for *T. ramosissima*. PR, RD, FDAR, SDAR, and TDAR refer to the pre-rainfall day, rainfall day, the first day following rainfall, the second day following rainfall, and the third day following rainfall, respectively. Daytime is defined from 06:00 to 20:00 (local time), and the ratio indicates the percentage of total daytime sap flow to total day value in pre-rainfall, rainfall and post-rainfall days.

Diurnal variations in mean half-hourly stem sap flow for *T. ramosissima* on the pre-rainfall day, rainfall day, and the three days following rainfall across the different rainfall categories are presented in **Figure 7**. The half-hourly stem sap flow on pre-rainfall day was consistently higher than that observed on the rainfall day, regardless of the rainfall category. The mean half-hourly stem sap flow on pre-rainfall and the rainfall day exhibited distinct diurnal patterns in both rainfall categories, with peak values occurring at around 12:00 (local time). Furthermore, the results shown that daytime stem sap flow (from 6:00 to 20:00, local time) on the pre-rainfall and rainfall day of *T. ramosissima* in the two rainfall categories accounted for 98.8% to 99.9% and 98.4% to 99.7% of the total whole-day pre-rainfall stem sap flow, respectively.

Compared to pre-rainfall conditions, there was non-significant variation in half-hourly stem sap flow during the three days following rainfall in rainfall category I and rainfall category II. Although the mean half-hourly stem sap flow increased after the rainfall events relative to the rainfall day, the proportion of daytime stem sap flow to total daily stem sap flow remained largely unchanged, regardless of rainfall category. After the rainfall events, the half-hourly stem sap flow rapidly recovered to its pre-rainfall status, with a peak at noon time within the two rainfall categories. The results showed that, in the three days after rainfall events, 98.2%-98.7% and 99.8%-99.9% of the total daily stem sap flow occurred during the day time in rainfall categories I and II, respectively.

## 4 Discussion

### 4.1 Controlling factors of daily stem sap flow dynamics in two rainfall categories

In accordance with the principles of basic plant physiology, it can be hypothesized that precipitation will exert a considerable influence on the sap flow dynamics of *T. ramosissima*. Nevertheless, the results of this study demonstrate that there is no statistically significant positive correlation between daily stem sap flow and SMC_0-40cm_ after precipitation (**Figures 5**, **6**) within the two rainfall categories, contrary to the initial hypothesis. This finding aligns with the “plus-reserve” hypothesis, which posits that smaller rainfalls only affects relatively minor ecological events (e.g., micro-fauna and mic-flora), whereas the larger rainfall affects the physiology and growth of plants (Schwinning and Sala, 2004). Plants do not usually respond directly to rainfall, but rather to the increase in SMC that it causes. The response thresholds of rainfall thresholds are often determined by the ability of plants to utilize soil moisture of different infiltration depth or duration (Schwinning and Sala, 2004). The result of previous studies has demonstrated that stem sap flow increases significantly with higher levels of rainfall (Zhao and Liu, 2010; Jian et al., 2016; Wang et al., 2020; Iqbal et al., 2021), as water is often the limiting factor in desert ecosystems, and positive feedback between SMC and stem sap flow is typically expected. Contrary to this finding, the result of our study indicate that the daily stem sap flow of *T. ramosissima* on pre-rainfall day was consistently higher than that on the rainfall day, irrespective of the rainfall category. Furthermore, there was a non-significant increase in daily stem sap flow in the three days following rainfall events, compared with the results observed in pre-rainfall and on the rainfall day. These results align with the conclusions of previous studies (Chen et al., 2014; Yan et al., 2018). And this uncertainty may be attributed to changes in atmospheric and soil conditions induced by rainfall events, which indirectly result in alterations to the unique physiological characteristics of vegetation. During the growing season, the SMC may be insufficient to meet atmospheric demand, particularly when Ta and VPD are highest, leading to suppressed stem sap flow. During the experimental period, a total of 63.8 mm of precipitation was recorded. Of this, 44% was of rainfall category I, while 56% was of rainfall category II. The results of this study show that the SMC at 0–40 cm depth exhibited a pronounced response to rainfall events; in comparison, the SMC at other depths remained largely unchanged. However, the finding of this study shown that rainfall category I does not exert an appreciable influence on available SMC at 0–40 cm depth. Despite an increase SMC at 0–40 cm depth and 40–160 cm depth from 0.11 cm^3^·cm^-3^ and 0.07 cm^3^·cm^-3^, respectively, prior to precipitation, to 1.65 cm^3^·cm^-3^ and 0.11 cm^3^·cm^-3^ on the first post-precipitation day, there was no corresponding increase in available SMC (0.00 cm^3^·cm^-3^and -0.13 cm^3^·cm^-3^, respectively) at the aforementioned depths in rainfall category II. Nevertheless, the recorded maximum rainfall was 11.3 mm (on 23 August) during the experimental period. More specifically, the SMC at depths of 5 cm, 10 cm, 20 cm and 40 cm have been re-analysed. Although the soil moisture content increased from 0.10 cm³·cm^−3^ to 0.13 cm³·cm^−3^ at a depth of 5 cm and from 0.09 to 0.10 cm³·cm^−3^ at a depth of 10 cm (**Supplementary Table S3**), the wilting point at these depths is 0.12 cm³·cm^−3^. These findings clearly demonstrate that the rainfall level during the experimental period was insufficient to significantly increase the available SMC at 0–40 cm depth.

The daily stem sap flow of *T. ramosissima* is strongly influenced by meteorological factors. When the VPD is high and accompanied with very low SMC, the stem sap flow of plants will decrease or even diminish, which leads to the die of plants. However, *T. ramosissima* is a phreatophyte xerophytic shrub that depends on deep soil moisture or groundwater (Liu et al., 2022). Even under conditions of extremely low SMC in both the 0–40 cm and 40–160 cm soil layers, it maintains normal physiological processes by accessing deep soil moisture or groundwater. Therefore, the sap flow of *T. ramosissima* is controlled by meteorological factors. On typically sunny days, stem sap flow increases as PAR and Ta rise, while RH decreases. This phenomenon may be attributed to the expansion of the potential gradient between leaf-to-air boundaries (Doyle et al., 2007; Huang et al., 2010). Nevertheless, it is anticipated that rainfall events will markedly escalate the RH and concurrently diminish Ta. It is expected that this change will result in a reduction in daily stem sap flow on the rainfall day and may be associated with the fact that stomata tend to close with the change in meteorological conditions and leaf water status (Granier, 1987; Irvine et al., 1998), which is in accordance with the results of previous studies (Zhao and Liu, 2010; Wang et al., 2020). Following the cessation of rainfall, an increase in PAR, Ta, and RH over the subsequent three days resulted in a marked increase in the daily stem sap flow of *T. ramosissima*. However, compared to pre-rainfall conditions, there was no notable change in SMC or available SMC following rainfall. Conversely, rainfall events result in a reduction in PAR and Ta, accompanied by an increase in RH, which contributes to the negative relationship between SMC_0-40cm_ and daily stem sap flow (**Figure 5G**).

### 4.2 The effects of rainfall on the daily stem sap flow dynamics of *T. ramosissima*

In general, native plants exhibit reduced responsiveness to environmental variation relative to invasive plants in resource-limited environments, particularly when plant functions are directly linked to resource conservation (Du et al., 2011; Funk, 2013; Ghimire et al., 2018). *T. ramosissima* is a native phreatophyte xerophytic shrub in the terminal area of the Shiyang River in northwest China, which may contribute to weakly response of sap flow dynamics to rainfall variability. The divergence between native and exotic plants may be attributed to differences in hydraulic root resistance, resource competition, and the competitive abilities of individual shrub (Ghimire et al., 2018; She et al., 2019). Under conditions of dry air, stomatal control over transpiration is intensified (Bernier et al., 2006). The prolonged exposure of plants to conditions of water scarcity results in a diminished responsiveness to alterations in the surrounding environment (Mcdowell et al., 2011; Xu and Zhou, 2011). Diminished sensitivity to environmental stress during drought reflects an adaptive regulation of transpiration that prioritizes water conservation (Zhang et al., 2019; Su et al., 2022). Meanwhile, Xu et al. (2009) employed the same method to measure the stem sap flow of irrigated *T. ramosissima* in the Tarim Desert Highway shelterbelt, located in the Taklimakan Desert. Their results showed that the annual and average daily water consumption of *T. ramosissima* were 1253.39 *kg* and 6.27 kg·d^−1^, respectively, which is extremely higher than the values obtained in our study (75.43 kg and 523.8 g·d^−1^, respectively). Daily stem sap flow of *T. ramosissima* with deep groundwater table (<9 m) remained relatively low during the experimental period, indicating that *T. ramosissima* adopted a conservation water use strategy. This physiological response appears to be mediated through progressive stomatal regulation in response to elevated atmospheric VPD. Such stomatal control mechanisms enable the maintenance of favorable plant water status above critical thresholds, thereby preventing xylem dysfunction through hydraulic failure and cavitation.

The disparate responses of the plant to specific rainfall thresholds were predominantly associated with the source of water on which plant survival was contingent (e.g., topsoil or deep soil water), in addition to the plants’ distinctive water-consumption patterns (Beatley, 1974; Ewers and Oren, 2003; Iqbal et al., 2021). For example, Yan et al. (2018) compared the stem sap flow dynamics of *Pinus tabuliaeformis* and *Betula albosinensis* under varying moisture conditions. The results showed that with increasing soil moisture content, the stem sap flow of *P. tabuliformis* increased significantly; in comparison, the stem sap flow of *B. albosinensis* remained predominantly stable. In addition, Jia et al. (2024) found that soil moisture is an important factor affecting stem sap flow. However, this study did not identify any rainfall thresholds that could significantly increase the stem sap flow of *T. ramosissima.* The daily sap flow of *T. Ramosissima* on the pre-rainfall day (22 August), the rainfall day (with the largest rainfall of 11.3 mm on 23 August), the first day after rainfall (24 August), the second day after rainfall (25 August), and the third day after rainfall (26 August) were 544.8 ± 95.6 g·d^−1^, 379.0 ± 95.7 g·d^−1^, 597.1 ± 110.0 g·d^−1^, 600.7 ± 108.8 g·d^−1^ and 682.8 ± 121.0 g·d^−1^ respectively. Although the daily stem sap flow on post-rainfall days appeared to increase visually compared to pre-rainfall levels, statistical analysis revealed no significant difference in sap flow after the precipitation (**Supplementary Figure S5**). Additionally, the diurnal variations in half-hourly stem sap flow and meteorological factors revealed that the peak time for VPD lagged behind that for stem sap flow of *T. ramosissima*, both before and after the rainfall events on 23 August (**Supplementary Figure S6**). In general, as atmospheric evaporation demand (PAR, Ta, and VPD) increases, the stem sap flow will gradually increase. When PAR and VPD reach their peaks, *T. ramosissima* close some stomata to prevent excessive water extraction from the trunk from xylem vessel embolism and the collapse of the hydrological conductive system of the xylem. Therefore, we are more inclined to believe that the hysteresis between VPD and stem sap flow is a form of self-protection for plant, mitigating water stress in a waterless environment. Moreover, Liu et al. (2022) found that *T. ramosissima* take up water mainly from deep soil moisture and groundwater (>90%). These results indicate that the variation in rainfall pattern are unlikely to significantly affect the daily stem sap flow of *T. ramosissima* via alteration in topsoil moisture. It can therefore be concluded that the level of rainfall recorded during the experimental period was insufficient to trigger a significant increase in stem sap flow in *T. ramosissima*.

## 5 Conclusion

This study explored the response of stem sap flow dynamics to two different rainfall categories. The result showed that rainfall events led to a reduction in daily stem sap flow by 46.5% and 29.5% across rainfall category I and II, respectively. Nonetheless, non- significant differences in daily stem sap flow were observed during the three days following rainfall events in comparison to pre-rainfall conditions, irrespective of rainfall category (*P*>0.05). Moreover, mean daily PAR, daily Ta, and daily VPD is the primary drivers of stem sap flow on rainfall and the subsequent three days. Additionally, diurnal variations in half-hourly stem sap flow during the three days post-rainfall showed non-significant deviations compared to pre-rainfall levels or those on the rainfall day. Thus, we conclude that impacts of rainfall variability on the stem sap flow of *T. ramosissima* are primarily driven by meteorological factors, independent of the rainfall category, rather than soil water content as initially hypothesized. We can therefore conclude that the impact of rainfall variability on the stem sap flow of *T. ramosissima* are primarily driven by meteorological factors, independent of the rainfall category, rather than soil water content as initially hypothesized. These findings provide critical insights into how plant stem sap flow responses to rainfall events in arid regions. These findings provide critical insights into plant stem sap flow responses to rainfall events in arid regions, highlighting the necessity of considering meteorological variables and rainfall category characteristics in such assessments.

## Data Availability

The original contributions presented in the study are included in the article/**Supplementary Material**. Further inquiries can be directed to the corresponding authors.
