# Supplementary material for: The response of the stem sap flow dynamics of Tamarix ramosissima to rainfall variability in the desert-oasis transition zone of Northwest China
Source: Front Plant Sci. 2025 Jul 23;16:1563166. doi: 10.3389/fpls.2025.1563166 (PMC12327095; doi:10.3389/fpls.2025.1563166)
Supplement: Supplementary file 1 [file DataSheet1.docx]

**The Supplementary Table 1**. Soil particle composition of 0-40 cm soil profile

| Depth (cm) | Clay (%) | Silt (%) | Sand (%) | Soil texture (USDA Soil Texture Classification System) |
| --- | --- | --- | --- | --- |
| 5 | 20.26±0.30 | 41.40±0.91 | 38.34±1.18 | Loam |
| 10 | 23.58±1.03 | 46.08±1.04 | 30.34±2.06 | Loam |
| 20 | 22.88±0.30 | 44.29±1.06 | 32.83±0.76 | Loam |
| 40 | 24.68±0.31 | 41.54±0.36 | 33.78±0.22 | Loam |

**The Supplementary Table 2.** Statistical information of daily maximum meteorological factors among two rainfall categories during the experimental period in 2020.

| Rainfall category |  | Daily Max PAR (μmol·m^-2^s^-1^) | Daily Max Ta (℃) | Daily Min RH (%) | Daily Max VPD (kPa) |
| --- | --- | --- | --- | --- | --- |
| Category Ⅰ | PR | 1840±115.7aA | 29.5±1.6aA | 24.5±2.9aA | 3.3±0.4aA |
|  | RD | 1714.1±161.7aA | 25.2±1.4bA | 31.7±3.0aA | 2.3±0.3bA |
|  | FDAR | 1991.0±49.2aA | 28.5±1.3aA | 19.2±2.0aA | 3.2±0.3aA |
|  | SDAR | 2046±65.4bA | 28.4±1.3aA | 18.6±2.5aA | 3.3±0.3aA |
|  | TDAR | 1941.5±61.4aA | 30.0±1.6aA | 15.4±1.7bA | 3.8±0.3aA |
| Category Ⅱ | PR | 1880.4±39.8aA | 28.7±0.8aA | 26.0±4.7aA | 2.9±0.3aA |
|  | RD | 1453.6±347.6aA | 25.0±1.6aA | 33.4±3.9aA | 2.2±0.3aA |
|  | FDAR | 2000±62.3aA | 26.0±1.7aA | 31.2±5.2aB | 2.4±0.4aA |
|  | SDAR | 1965.6±53.2aA | 29.9±1.1bA | 18.5±2.7bA | 3.5±0.3bA |
|  | TDAR | 1954.8±207.7aA | 30.4±1.8bA | 17.2±3.3bA | 3.7±0.5bA |

**Note:** PR, RD, FDAR, SDAR, and TDAR are pre-rainfall, the rainfall day, the first day after rainfall, the second day after rainfall, and the third day after rainfall. The average values mentioned above are expressed as the means ± standard error. Different upper-case letters indicate significant differences in meteorological factors on the same day among the different rainfall categories (t-test, *P*<0.05). Different lower-case letters indicate significant differences in meteorological factors during pre-rainfall, rainfall and after rainfall in the same rainfall category (ANOVA, *P*<0.05).

**The Supplementary Table 3.** The variation of soil moisture at 0-40 cm soil layer before and after rainfall (on 23 August)

| Depth (cm) | PR (cm^3^·cm^-3^) | RD (cm^3^·cm^-3^) | FDAR (cm^3^·cm^-3^) | SDAR (cm^3^·cm^-3^) | TDAR (cm^3^·cm^-3^) |
| --- | --- | --- | --- | --- | --- |
| 5 | 0.10 | 0.11 | 0.12 | 0.13 | 0.13 |
| 10 | 0.09 | 0.09 | 0.10 | 0.10 | 0.10 |
| 20 | 0.11 | 0.11 | 0.11 | 0.11 | 0.11 |
| 40 | 0.12 | 0.12 | 0.12 | 0.12 | 0.12 |

**Note:** PR, RD, FDAR, SDAR, and TDAR refer to pre-rainfall (8/22), the rainfall day (8/23), the first day after rainfall (8/24), the second day after rainfall (8/25), and the third day after rainfall (8/26), respectively.

*
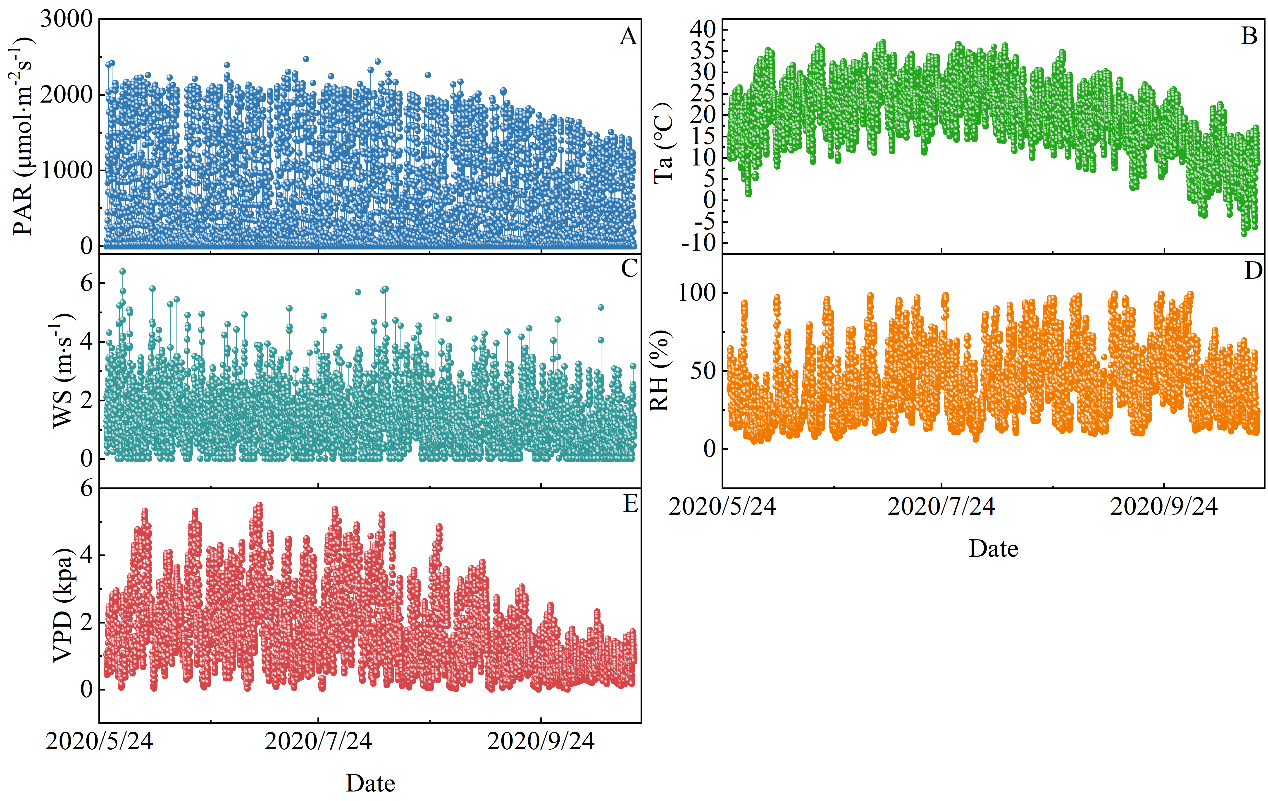
*

**The Supplementary Fig.1.** Changes in meteorological factors during the experimental period in 2020. (A) half hourly photosynthetically active radiation (PAR), (B) half hourly air temperature, (C) half hourly wind speed (WS), (D) half hourly relative humidity (RH), and (E) half hourly vapor pressure deficit (VPD) during the experimental period in 2020.


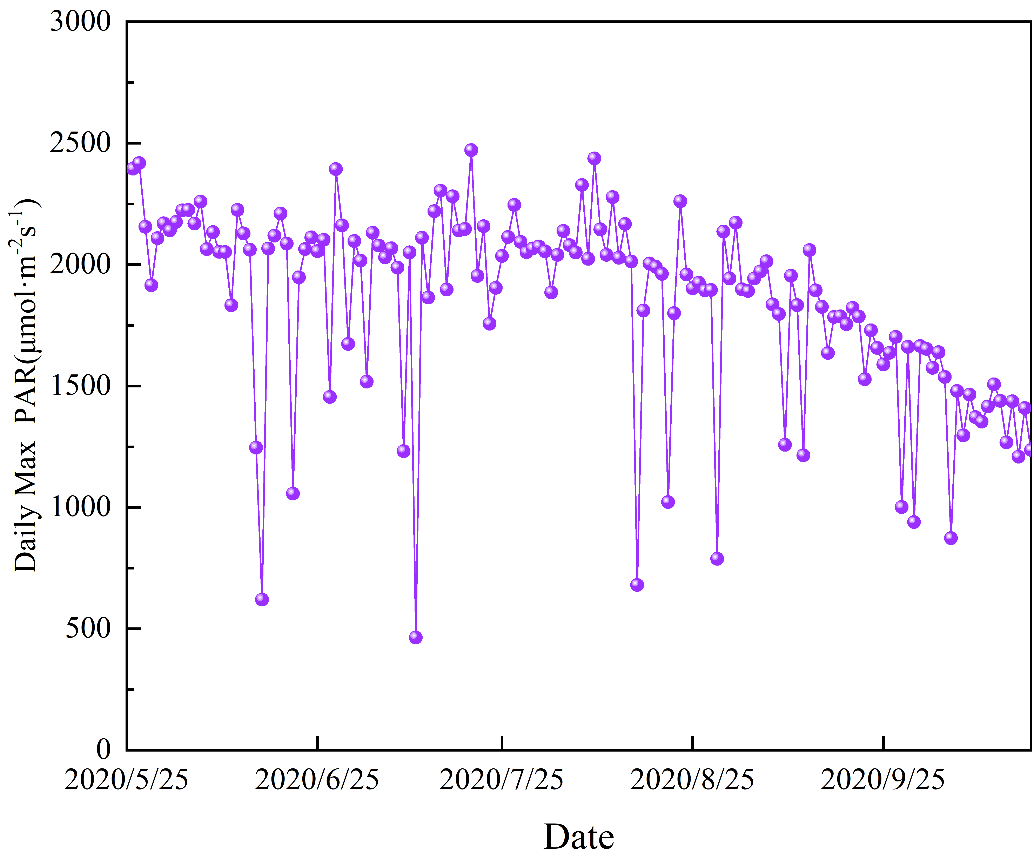


**The Supplementary Fig.2.** Changes in daily maximum PAR during the experimental period in 2020.

*
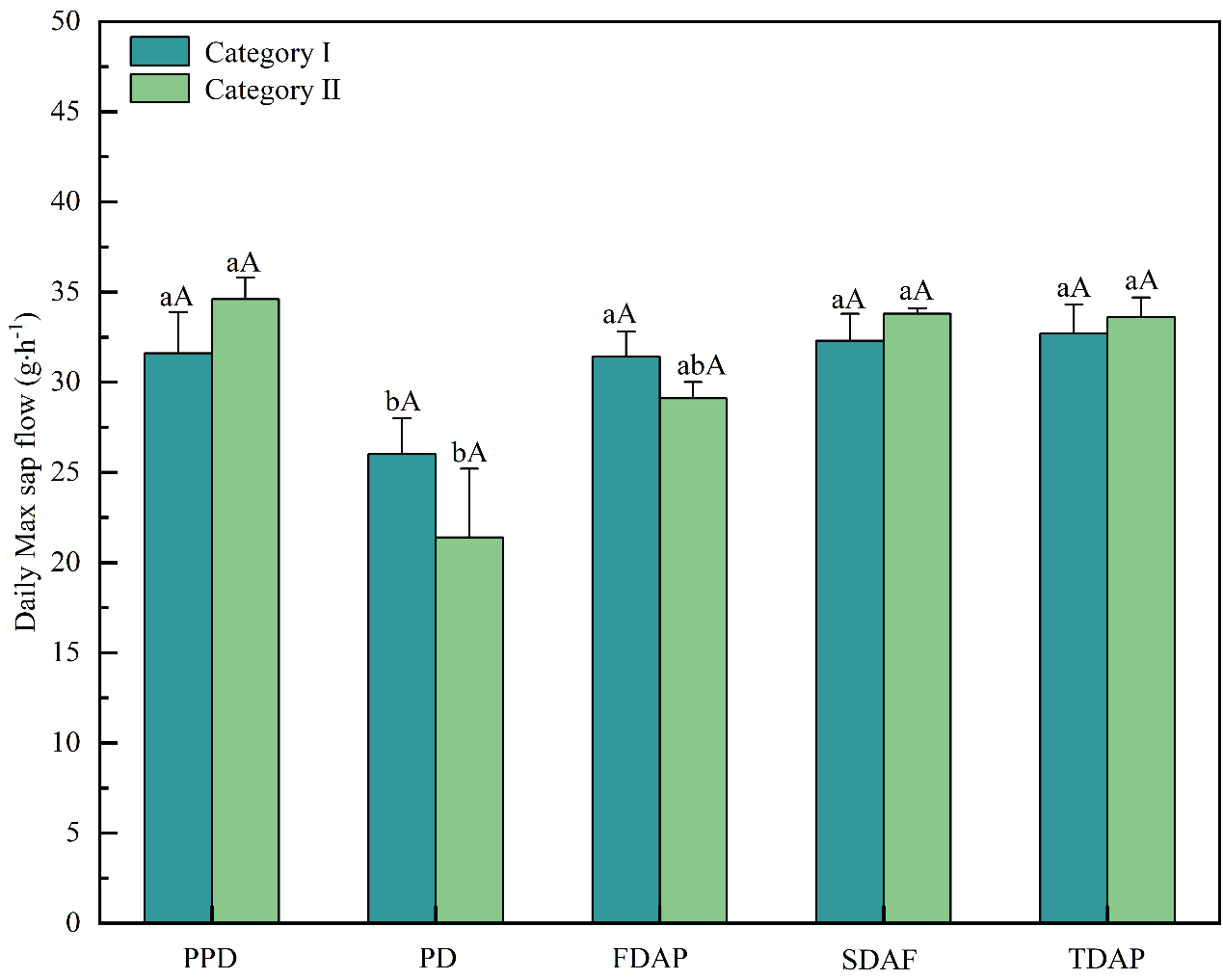
*

**The Supplementary Fig.3.** Comparison of the daily max sap flow among the pre-rainfall, rainfall, and post-rainfall periods among different rainfall categories during the experimental period in 2020 for *T.ramosissima*. **Note:** PR, RD, FDAR, SDAR, and TDAR refer to pre-rainfall, the rainfall day, the first day after rainfall, the second day after rainfall, and the third day after rainfall, respectively. Different uppercase letters denote significant differences in daily max sap flow on the same day among category Ⅰ and category Ⅱ (*P*<0.05). Different lowercase letters signify significant differences in daily max stem sap flow among the pre-rainfall, rainfall, and post-rainfall periods within the same rainfall category (*P*<0.05).

*
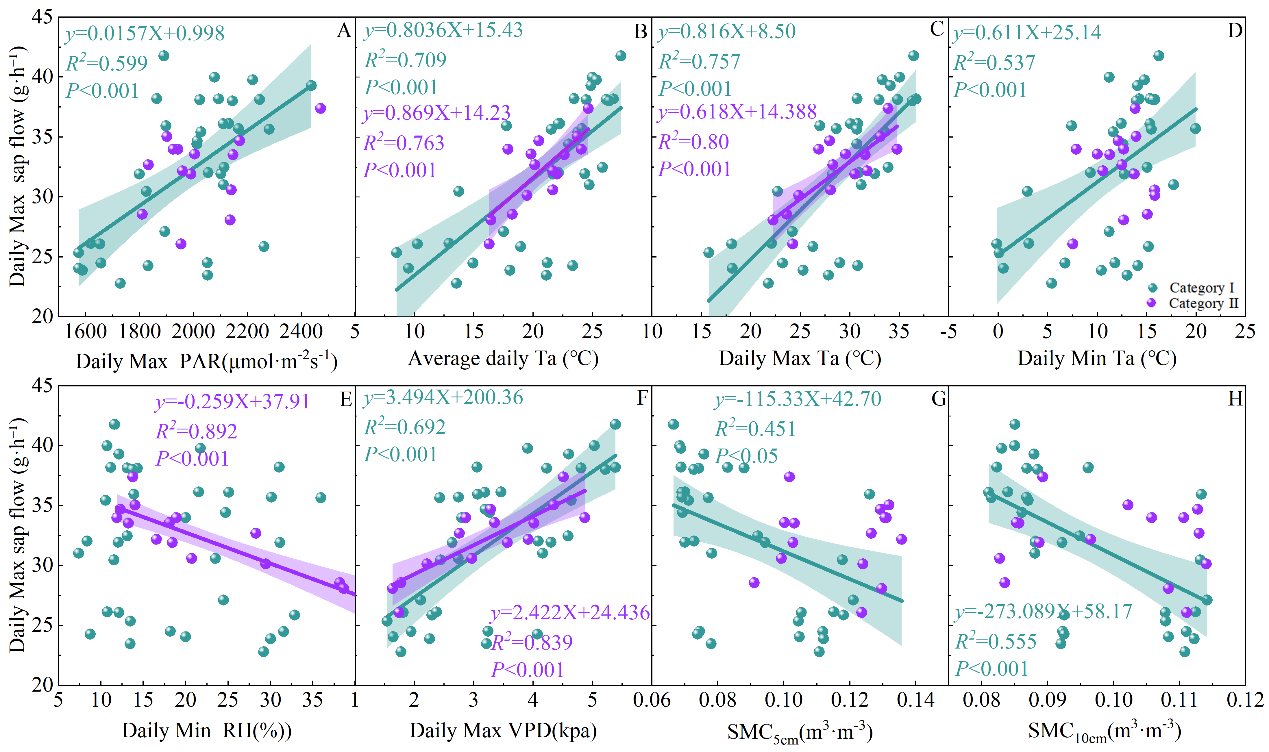
*

**The Supplementary Fig.4.** Relationship between daily maximum stem sap flow and daily maximum PAR, mean daily Ta, daily maximum Ta, daily minimum Ta, daily minimum RH, daily maximum VPD, SMC_5cm_, and SMC_10cm_ for *T.ramosissima* in the following three days of rainfall events of two rainfall categories during the experimental period in 2020.


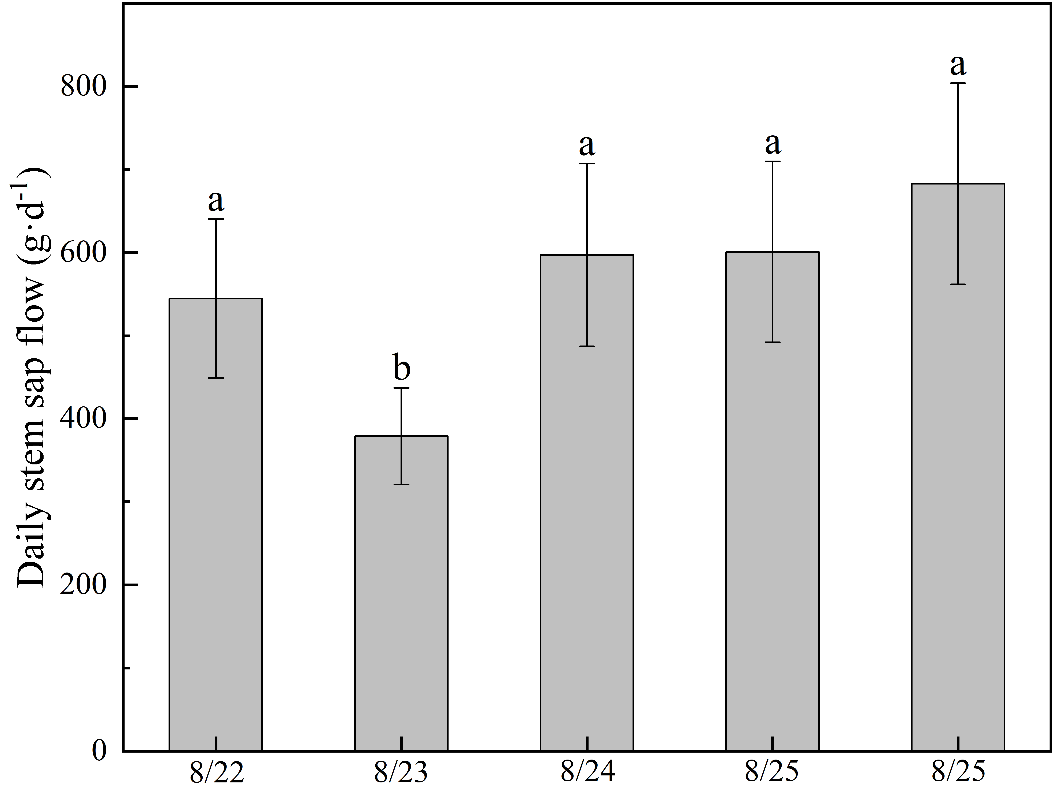


**The Supplementary Fig. 5.** The comparison of mean daily stem sap flow before and after the 23 August in 2020


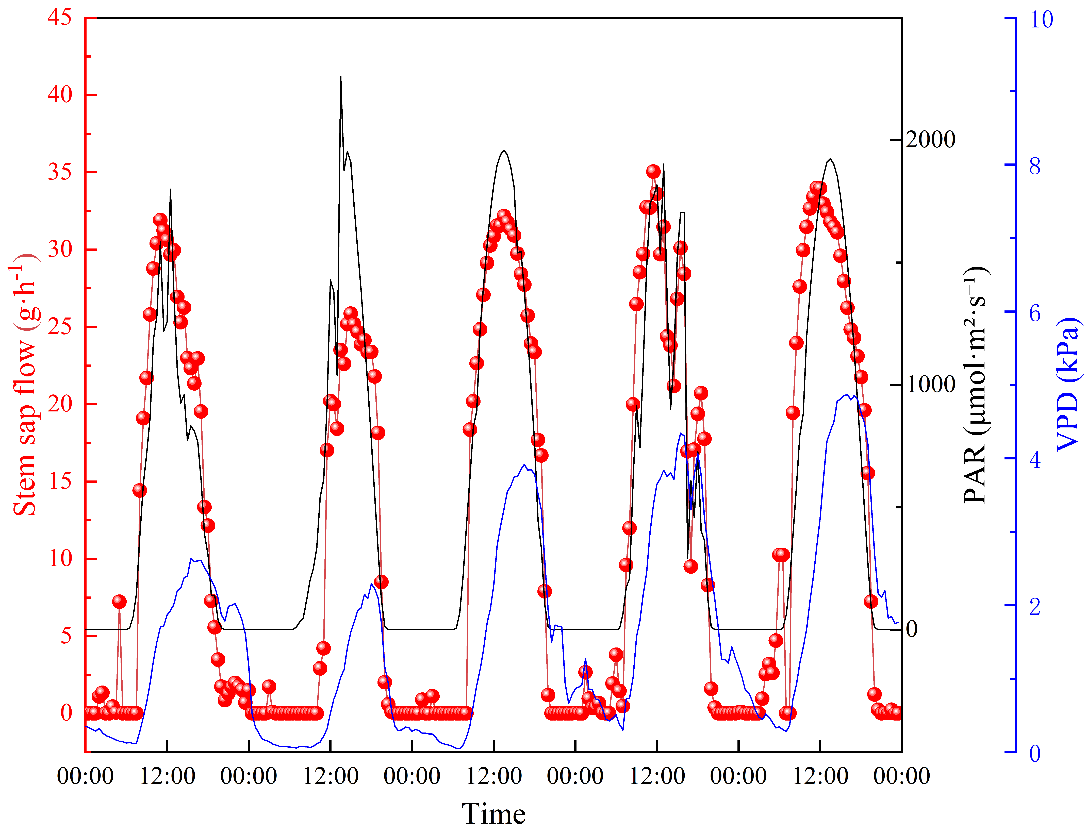


**The Supplementary Fig. 6.** The diurnal variations of half-hourly sap flow, PAR, and VPD before and after the 23 August in 2020
